# Innovative Pest Management Strategies in Phytoplasma Insect Vectors Control

**DOI:** 10.3390/plants15111664

**Published:** 2026-05-29

**Authors:** Adalbert Balog, László Hevér, Artúr Botond Csorba

**Affiliations:** 1 Department of Horticulture, Faculty of Technical and Human Sciences, Sapientia Hungarian University of Transylvania, Aleea Sighișoarei 2, Corunca, 540485 Târgu Mureș, Romania; 2 Department of Agrobiology, Institute of Biology, Faculty of Sciences, University of Pécs, Ifjúság Str. 6, 7624 Pécs, Hungary; hever.laszlo@pte.hu

**Keywords:** biological control, IPM control, leafhoppers, planthoppers, psyllids, semiochemicals

## Abstract

Phytoplasmas are obligate intracellular plant pathogens that cause numerous severe plant diseases in agricultural crops worldwide. Phytoplasmas are transmitted primarily by phloem-feeding insect vectors such as leafhoppers, planthoppers, and psyllids, transmitting the pathogen between infected and healthy plants. Conventional management strategies mainly consider chemical insecticides, which often lead to environmental concerns, insect resistance, and limited long-term effectiveness. Consequently, innovative and sustainable approaches have emerged for managing phytoplasma vector populations and interrupting disease transmission only in the last few years. Several disease outbreaks, i.e., the expansion of the American grapevine leafhopper (*Scaphoideus titanus*) in Europe, underlined the necessity of developing new and innovative technologies for phytoplasma control. This article reviews classical and innovative pest management strategies against insect vectors of phytoplasma, and a detailed comparison of the efficiencies between methods is presented. The integration of these advanced approaches within integrated pest management (IPM) frameworks offers promising prospects for sustainable crop protection and phytoplasma disease mitigation.

## 1. Introduction

Phytoplasmas are pathogens that have lost many parts of their genome, and their functions are more similar to those of viruses [1,2]. They are considered to belong to the class Mollicutes, which colonize plant phloem tissues, causing numerous plant diseases such as witches’ broom, phyllody, yellowing, and decline. These pathogens infect a wide range of crops, including grapevine, fruit trees, cereals, and vegetables [2]. Unlike many other plant pathogens, phytoplasmas are primarily transmitted by phloem-feeding insect vectors [3,4].

The most important insect vectors include species of leafhoppers (Cicadellidae), planthoppers (Fulgoroidea), and psyllids (Psylloidea) [5,6,7]. These insects acquire phytoplasmas while feeding on infected plants (crop plants and weeds), after which the pathogens multiply within the insect body and eventually colonize the salivary glands, enabling transmission to healthy plants during feeding [7].

Traditional control methods to control phytoplasma transmission focus mainly on insecticide application to reduce vector populations; however, this approach has several limitations, including pesticide resistance, environmental pollution, and negative impacts on beneficial organisms [8]. Therefore, modern research and innovative approaches toward innovative and sustainable pest management strategies that specifically target vector biology, behavior, and ecological interactions are increasingly necessary.

## 2. Biology of Phytoplasma Insect Vectors

Phytoplasma transmission is highly specialized and occurs through persistent propagative interactions between the pathogen and insect vectors. Once ingested from infected plants, phytoplasmas cross the insect gut barrier, circulate through the hemolymph, and colonize the salivary glands before being transmitted during subsequent feeding events [9]. The most important vector species include:

- *Scaphoideus titanus*—vector of grapevine Flavescence dorée phytoplasma.
- *Hyalesthes obsoletus*—vector of stolbur phytoplasma.
- *Macrosteles quadripunctulatus*—vector of several phytoplasma diseases.
- *Cacopsylla picta* and *Cacopsylla melanoneura*—vectors of the apple proliferation phytoplasma.

Following ingestion from infected plant phloem, phytoplasmas must overcome multiple anatomical and immunological barriers within the insect vector. Initially, they traverse the midgut epithelium, enter the hemocoel, and subsequently colonize secondary tissues, particularly the salivary glands, which are essential for successful transmission during feeding [9]. This internal transfer of the pathogen is highly selective and involves molecular recognition processes between phytoplasma surface proteins (e.g., antigenic membrane proteins) and insect cellular components, including cytoskeletal proteins [10]. Additionally, transcriptomic analyses of vector insects revealed that pathogen acquisition induces significant gene expression changes in the gut and salivary gland tissues of vectors, particularly in genes related to immunity and membrane transport, supporting the concept of an active and regulated infection process rather than passive circulation [11].

Only a limited number of phytoplasma-infected insects become competent vectors, emphasizing the specificity of these interactions [11]. The latency period, defined as the time required for phytoplasma multiplication and migration to salivary glands, is influenced by environmental conditions such as temperature, which modulates both pathogen replication and vector feeding behavior [5,10]. Also, differences between species can occur, but most of these mechanisms are not fully understood. Some details about specific phytoplasma vectors are presented below.

*S. titanus* is the primary vector of *Flavescence dorée* phytoplasma (‘*Candidatus Phytoplasma vitis*’) in European vineyards [12]. This leafhopper exhibits a high degree of vector specificity and efficiency, making it a major vector of this disease. Recent studies highlight the stability of its associated microbiome across developmental stages, suggesting a strongly regulated internal environment that may influence vector competence and phytoplasma colonization dynamics [13]. The pathogen is consistently detected in key organs such as the midgut, fat body, and salivary glands of the vector, confirming its propagative life cycle [13]. Beyond its established role as the vector of Flavescence dorée phytoplasma, recent studies have provided deeper insights into its internal biology. Metatranscriptomic and microbiome analyses revealed that *S. titanus* harbors a stable core microbiome, including *Candidatus Sulcia muelleri* and *Cardinium*, which may influence vector competence and pathogen persistence [11]. More recent work demonstrates that host plant identity significantly alters the bacterial community composition of *S. titanus*, suggesting indirect effects on phytoplasma transmission efficiency through ecological interactions [14]. In addition, experimental studies show that phytoplasma transmission efficiency can be modulated by external treatments affecting vector feeding and acquisition processes, further confirming the dynamic nature of vector competence [15].

The planthopper *H. obsoletus* is the principal vector of ‘*Candidatus Phytoplasma solani*’ (stolbur phytoplasma), responsible for Boi’s noir disease in grapevine. This species demonstrates strong ecological specialization, with distinct host plant associations (e.g., *Convolvulus arvensis*, *Urtica dioica*), which shape its epidemiological role [16]. Its polyphagous feeding behavior facilitates pathogen acquisition from reservoir weed plants and subsequent transmission to crops. Moreover, only a subset of infected individuals are capable of transmission, reflecting the importance of specific vector–pathogen compatibility mechanisms [10]. Field-based evidence demonstrates that a substantial proportion of individuals (up to ~40%) can carry phytoplasma under natural conditions, supporting its epidemiological importance in vineyard systems [17].

*Macrosteles quadripunctulatus* is a polyphagous leafhopper involved in the transmission of several phytoplasmas, including ‘*Candidatus Phytoplasma solani*’ and ‘*Candidatus Phytoplasma asteris*’. Although many insect species may harbor the above mentioned phytoplasmas, relatively few, including *M. quadripunctulatus,* have been experimentally confirmed as effective vectors. Its role highlights the importance of vector specificity despite widespread phytoplasma presence in insect communities [10].

*C. picta* and *C. melanoneura* psyllid species are the main vectors of the *apple proliferation phytoplasma* (‘*Candidatus Phytoplasma mali*’). Studies on disease dynamics demonstrate that pathogen accumulation within the insect varies temporally and is linked to transmission efficiency. The successful colonization of salivary glands is a critical determinant of vector competence, and fluctuations in phytoplasma titre within the insect body directly affect epidemiological outcomes [18]. Quantitative studies further demonstrate that vector capacity to infect plants correlates with pathogen thresholds, suggesting that transmission is not only dependent on infection status but also on pathogen density within specific tissues [19].

## 3. Limitations of the Conventional Management of Phytoplasma Vector

The management of phytoplasma-associated diseases has considered a combination of chemical, agronomic, and sanitary measures, including the application of insecticides targeting vector populations, the removal of infected plants, the use of certified phytoplasma-free propagation plant material, and general crop hygiene practices.

While these approaches can reduce disease incidence in the short term, new scientific evidence indicates that they are often insufficient for sustainable long-term control [20].

### 3.1. Chemical Insecticides and Resistance Development

The use of chemical insecticides remains the primary strategy for controlling phytoplasma insect vectors such as leafhoppers, planthoppers, and psyllids. In viticulture, for example, repeated treatments targeting *S. titanus* are widely implemented to control Flavescence dorée [21].

However, intensive and repeated insecticide use has led to increasing concerns about resistance development, reduced efficacy, and the disruption of agroecosystem balance. Recent studies highlight that vector populations exposed to frequent treatments may develop physiological resistance or behavioral avoidance, compromising control efficiency [22]. Other field studies also presented that even intensive insecticide applications cannot prevent phytoplasma spread because transmission can occur rapidly after vector feeding begins. In particular, insecticides may reduce vector abundance within crops but cannot prevent initial infection events caused by incoming infective insects [23]. Resistance development has been extensively documented across insect vectors and is now recognized as a major factor reducing control efficacy. Importantly, insecticide resistance can also influence vector competence, potentially altering transmission dynamics in unpredictable ways [24].

### 3.2. Environmental Contamination and Non-Target Effects

Another major limitation of conventional management is the environmental impact of chemical control strategies [5]. Broad-spectrum insecticides can contaminate soil and water systems and negatively affect non-target organisms, including pollinators and natural enemies of pests [12]. Recent assessments emphasize that insecticide-based control of phytoplasma vectors can significantly disrupt beneficial arthropod communities (especially pollinators), reducing ecosystem services such as biological control and pollination [5,12,25].

## 4. New Innovative Strategies to Control Phytoplasma Insect Vectors

A fundamental limitation of conventional management is that its effects hardly achieve complete disease suppression. Based on the presented issues, it is timely to assess and develop new innovative methodologies for phytoplasma vector control. Phytoplasma pathosystems are characterized by complex epidemiological networks, involving multiple host plants, vector species, and environmental factors. Earlier and recent studies consistently emphasize that no single effective control strategy exists for phytoplasma diseases, and current approaches provide only partial and temporary mitigation [26]. As a result, innovative technologies are being explored to improve vector management while minimizing environmental impact, and these can include modern genetic knowledge, and also (if possible), AI-generated tools to control phytoplasma spread by vectors.

### 4.1. Genetic Control and Genome Editing

These strategies aim to manipulate insect populations at the genetic level, either by reducing their abundance (population suppression) or by altering their ability to transmit phytoplasmas (population replacement). Unlike conventional approaches, these methods target the biological determinants of vector competence and population dynamics, offering potentially sustainable and species-specific solutions [27].

These strategies are increasingly enabled by gene drive systems, which bias inheritance and allow engineered traits to spread through populations at rates exceeding Mendelian expectations [28]. Recent advances in genome editing have significantly accelerated this field by enabling precise, site-specific modifications in insect genomes, overcoming limitations of earlier transgenic or mutagenesis-based approaches [28,29]. Recent studies have presented that phytoplasma infection interferes with endocrine and post-transcriptional regulatory pathways in insect vectors, particularly those associated with juvenile hormone (JH) signaling and miRNA-mediated developmental control. Alteration of JH homeostasis may influence vector maturation, fecundity, longevity, and feeding behavior, thereby affecting transmission efficiency [30]. In parallel, phytoplasma-responsive miRNAs have been implicated in the regulation of developmental and reproductive pathways through modulation of hormone-responsive genes and ecdysone/JH-associated signaling networks [31].

These molecular changes are consistent with the reproductive suppression frequently observed in infected hosts and vectors, where phytoplasma effectors manipulate hormonal balance and developmental programs to favor pathogen persistence and dispersal. Importantly, these pathways represent promising molecular targets for next-generation vector management strategies, including RNA interference (RNAi), miRNA-based silencing approaches, and the disruption of vector endocrine signaling to reduce phytoplasma acquisition and transmission [30,32,33].

### 4.2. Population Suppression and Population Replacement Strategies

Population suppression aims to reduce or eliminate vector populations, thereby lowering phytoplasma transmission pressure [3].

CRISPR/Cas-based gene drives are currently the most promising tools in this category. These systems function by targeting genes essential for reproduction (e.g., female fertility genes) for viability (lethal gene disruption) as well as for sex determination (sex ratio distortion). CRISPR-mediated disruption of reproductive genes can result in high levels of sterility or population collapse within a few generations, especially when coupled with gene drive mechanisms that ensure rapid spread of the modification [34,35,36].

In contrast to suppression, population replacement aims to transform vector populations into non-competent carriers of phytoplasmas while maintaining their ecological role. Gene drive systems are essential in this context, as they enable the rapid spread of anti-pathogen traits throughout natural populations [37]. Recent studies emphasize that genome editing can be used not only to introduce refractory traits but also as a functional genomics tool to dissect vector pathogen interactions, thereby enabling targeted intervention strategies [38]. Despite their promise, genetic control strategies face several critical challenges: these include the evolution of resistance to gene drives or edited traits; incomplete knowledge of vector genomics, particularly in non-model species such as phytoplasma vectors; technical constraints in delivering genome editing tools across diverse insect taxa; and regulatory, ethical, and ecological concerns, particularly regarding gene drive release [39].

### 4.3. Antiviral and Pathogen-Interference Chemistries

Modern antiviral and pathogen-interference mechanisms are emerging as promising tools for reducing vector competence and limiting phytoplasma transmission in sustainable crop protection systems [40].

Recent advances in antiviral agrochemicals and molecular inhibitor design have focused on targeting pathogen-derived effectors, vector–pathogen molecular interactions, and host signaling pathways involved in colonization and transmission [41].

Rational agrochemical development, supported by structural biology, computational modeling, and effector protein characterization, enables the identification of highly specific inhibitory compounds capable of disrupting pathogen persistence without broad ecological [42].

In phytoplasma-associated pathosystems, interference with the effector-mediated manipulation of plant defense and vector attraction represents a particularly attractive strategy for next-generation disease management. Furthermore, mechanistic antiviral development integrating RNA-based technologies, small-molecule inhibitors, and precision-targeted bioactive compounds may provide durable and environmentally compatible alternatives to conventional insecticide-dependent control programs [43,44,45,46].

### 4.4. Sterile Insect Technique (SIT)

The Sterile Insect Technique (SIT) is a species-specific, autocidal pest control method that involves the mass rearing, sterilization, and release of male insects to suppress wild populations, mostly used for Diptera control [47,48]. Sterile males compete with wild males for mating, resulting in infertile eggs and a progressive decline in population size [49]. The SIT has been successfully implemented against several major agricultural pests, particularly Dipteran species such as fruit flies, and is increasingly explored as a tool for managing insect vectors of plant pathogens [50]. Ionizing radiation induces dominant lethal mutations in germ cells, rendering males sterile without significantly affecting their ability to mate. However, excessive radiation doses can reduce insect fitness, creating a trade-off between sterility and competitiveness [47]. Recent studies have focused on improving SIT efficiency through genetic sexing strains, which enable male-only releases and enhance control efficacy, as well as through integration with other control strategies within area-wide pest management programs [51].

Studies on hemipteran vectors—such as leafhoppers and psyllids—suggest that SIT could be adapted to these systems, although practical implementation remains limited; we do not know if sterilized individuals can or cannot transmit the pathogens. One major constraint is that many vector species have complex life cycles, low fecundity, or are difficult to mass rear, which complicates SIT deployment [52,53].

While SIT has not yet been widely deployed against phytoplasma vectors, its conceptual applicability is clear, particularly in systems where a single dominant vector species drives disease epidemiology, populations are geographically constrained, and mass rearing can be optimized. However, given the biological and epidemiological characteristics of phytoplasma pathosystems, SIT is likely to be most effective when combined with complementary approaches, such as habitat management or emerging genetic control strategies.

### 4.5. Behavioral Control Using Semiochemicals

The manipulation of insect behavior through semiochemicals represents a promising and environmentally sustainable strategy for managing vectors of plant pathogens, including phytoplasmas [54].

Semiochemicals—chemical signals that mediate interactions between organisms—can be exploited to interfere with host location, feeding behavior, aggregation, and reproduction of insect vectors [55].

Compared to conventional insecticides, these approaches are species-specific and can reduce non-target effects while integrating well into sustainable pest management [56].

Phytoplasma vectors, such as leafhoppers, planthoppers, and psyllids, rely heavily on plant-derived volatile organic compounds (VOCs) and contact cues to locate suitable hosts. Importantly, phytoplasma infection often alters plant physiology, leading to changes in volatile emission profiles that can influence vector behavior [55].

Several studies demonstrate that infected plants may become more attractive to vectors, thereby enhancing pathogen spread. For example, phytoplasma-induced changes in host plant volatiles have been shown to increase vector attraction and feeding activity, facilitating transmission [57,58]. More recent work confirms that these pathogen-mediated manipulations represent a form of extended phenotype, where the pathogen indirectly modifies vector behavior through plant signaling pathways [59].

Sex pheromones represent another class of semiochemicals with potential applications in vector control. In leafhoppers and related hemipterans, substrate-borne vibrational signals often complement or replace airborne pheromones, complicating direct application. However, research has shown that chemical and vibrational cues can be integrated to interfere with mating communication systems [60]. While pheromone-based mating disruption is less developed for phytoplasma vectors compared to Lepidoptera, it remains a promising area for future research.

The manipulation of plant-emitted semiochemicals can also be achieved indirectly through induced plant resistance. For instance, treatment with elicitors such as jasmonic acid or salicylic acid can modify plant volatile emissions, making them less attractive or even repellent to insect vectors. Studies indicate that induced changes in VOC profiles can significantly alter vector preference and feeding behavior, potentially reducing pathogen transmission [61].

### 4.6. Nanotechnology-Based Crop Protection

Nanotechnology-based crop protection has emerged as a promising new approach to improve the efficacy, specificity, and sustainability of plant disease and pest management. Nanoparticles (NPs), including metallic (e.g., silver, zinc oxide) and polymer-based systems, exhibit intrinsic antimicrobial and insecticidal properties. These materials can disrupt microbial cells through membrane damage, oxidative stress, and interference with metabolic processes [62].

Nanotechnology offers opportunities to intervene at multiple levels of the phytoplasma disease cycle, such as at the plant level: enhanced resistance through nano-delivered elicitors or antimicrobial agents; vector level: improved delivery of insecticides or RNAi targeting vector physiology; and at the environmental level: controlled release formulations reducing chemical inputs. Recent studies emphasize that nanotechnology can improve the precision of pest and disease management, aligning with sustainable agriculture goals [63].

In plant protection, nanoparticles have been shown to inhibit a broad range of pathogens, including bacteria and fungi, and to affect insect physiology and survival. Although phytoplasmas are not directly targetable due to their phloem-restricted lifestyle, nanoparticle-based treatments may indirectly reduce disease by affecting vector survival or feeding behavior [64,65].

## 5. Symbionts and Their Role in Phytoplasma Vector Control

Recent research increasingly emphasizes the role of endosymbiotic bacteria in modulating vector competence. In species such as *H. obsoletus* and *S. titanus*, stable symbiotic communities (e.g., *Candidatus Sulcia*, *Cardinium*) are localized in key tissues, including the gut and salivary glands. These symbionts may influence phytoplasma acquisition, replication, and transmission either through immune modulation or competition within host tissues [14].

Symbionts localized in critical tissues such as the gut, hemolymph, or salivary glands are particularly suitable targets, as these are the main sites of phytoplasma passage and multiplication within the insect [66,67].

Phytoplasma vectors, including leafhoppers, planthoppers, and psyllids, harbor complex microbiomes composed of obligate and facultative symbionts [47,48]. Among the most common are *Candidatus Sulcia muelleri* (primary symbiont), *Nasuia* spp. co-obligate symbionts and the facultative symbionts such as *Cardinium*, *Wolbachia*, and *Rickettsia*. These symbionts play essential roles in host nutrition, development, and immunity. Importantly, recent studies have shown that microbiome composition can influence vector competence, affecting phytoplasma acquisition and transmission efficiency [11]. In plant pathogen systems, early experimental work has demonstrated that symbiont manipulation can alter vector competence, although applications specifically targeting phytoplasmas remain limited [68].

Microbiome-based disease management is an emerging strategy that leverages the complex microbial communities associated with plants and insect vectors to reduce pathogen transmission and disease development. In phytoplasma pathosystems, both the plant microbiome and the insect vector microbiome play critical roles in shaping infection dynamics, offering novel targets for intervention [69]. Increasing evidence suggests that the microbiome can significantly affect vector competence, modulating phytoplasma acquisition, replication, and transmission [70].

Metatranscriptomic analyses in vectors such as *S. titanus* demonstrate that microbial communities are metabolically active and interact with host pathways involved in immunity and nutrient exchange, potentially influencing phytoplasma persistence [11]. Similarly, broader studies on insect–microbiome interactions highlight that symbionts can regulate pathogen colonization through immune priming, niche competition, and metabolic interference [71].

### Plant Microbiome and Disease Suppression

The plant-associated microbiome, particularly the phyllosphere and rhizosphere communities, also plays a role in modulating phytoplasma infections. Although phytoplasmas are restricted to phloem tissues, indirect effects mediated by the broader plant microbiome can influence disease severity and vector attraction. Recent studies emphasize that microbiome composition can affect plant volatile emissions, thereby altering vector behavior and transmission dynamics [72,73]. Naturally derived, eco-compatible biopesticides and semiochemical-based approaches are increasingly considered to be promising alternatives to synthetic insecticides for sustainable phytoplasma vector management. Recent advances in plant-derived bioactive compounds, including terpenoids, alkaloids, flavonoids, and essential oils, have demonstrated significant repellent, antifeedant, insecticidal, and behavioral-modifying effects against hemipteran insect vectors [74]. In parallel, semiochemical-based strategies exploiting volatile organic compounds (VOCs), pheromones, and host-derived signaling molecules have shown potential to interfere with vector host selection, feeding preference, and pathogen transmission dynamics. These environmentally compatible approaches may reduce pesticide dependence while preserving beneficial arthropod communities and minimizing ecological disruption. Furthermore, the integration of phytochemicals with RNAi technologies, nanodelivery systems, and precision ecological engineering represents an emerging direction for next-generation sustainable vector-control strategies in phytoplasma pathosystems [75].

## 6. Biological Control for Phytoplasma Vector Control

Biological control represents a key component of sustainable pest management and offers a promising alternative or complement to chemical strategies for managing insect vectors of phytoplasmas [76].

### 6.1. Natural Enemies of Phytoplasma Vectors

Phytoplasma vectors, particularly hemipterans such as leafhoppers, planthoppers, and psyllids, are subject to regulation by a diverse community of natural enemies, including predators (e.g., spiders, lacewings, predatory bugs), parasitoids (mainly Hymenoptera, such as *Anagrus* spp.), and entomopathogenic microorganisms (fungi, bacteria, viruses). Among these, egg parasitoids of the genus *Anagrus* (Hymenoptera: Mymaridae) are particularly important in regulating leafhopper populations, including vectors such as *S. titanus*. Field studies in vineyards have shown that *Anagrus* spp. can significantly reduce egg survival, contributing to natural population suppression [77].

### 6.2. Entomopathogenic Fungi and Microbial Control

Entomopathogenic fungi are among the most studied biological control agents for hemipteran vectors. Species such as the *Beauveria bassiana* and *Metarhizium anisopliae* have demonstrated effectiveness against a wide range of sap-feeding insects [78].

These fungi infect hosts through the cuticle, proliferate internally, and ultimately cause mortality. Experimental studies indicate that entomopathogenic fungi can significantly reduce vector populations and may also affect feeding behavior and longevity, thereby indirectly reducing phytoplasma transmission [79,80]. More recent work highlights the potential for integrating fungal biocontrol agents into IPM systems, although field efficacy can be influenced by environmental factors such as temperature, humidity, and UV exposure [81].

### 6.3. Conservation Biological Control

Conservation biological control focuses on enhancing the activity and effectiveness of naturally occurring enemies through habitat management and reduced pesticide use. In vineyard and orchard systems, increased habitat complexity has been associated with a higher abundance of parasitoids and predators, which can contribute to the suppression of vector populations [82].

## 7. Integrated Pest Management (IPM) for Phytoplasma Vector Control

Effective management against phytoplasma vectors requires an integrated approach with multiple methods that include cultural, chemical, and biological control measures [83].

However, the IPM methodology is less focused on phytoplasma vector control; nevertheless, the key components of using IPM methods include the use of certified disease-free planting material. No or limited information exists about these possibilities; i.e., only a few studies have tested the susceptibility of the grapevine or tomato varieties in these directions. Previous research has demonstrated that a decreasing gradient of infected grapevines occurs from the edges to the interior of vineyards [84,85]. In such cases, the less susceptible cultivars (if there are data on susceptibility) can be planted along the edge rows to reduce the risk of phytoplasma spread [86].

The monitoring of vector populations and habitat and ecological management are crucial to understanding new vector species (i.e., *S. titanus*—vector of grapevine Flavescence dorée phytoplasma), alternative host plants, overwintering stages, and habitat preferences. Integrating these approaches improves sustainability and reduces reliance on chemical insecticides [86,87]. Previous data demonstrated, for example, that *S. titanus* prefers to populate the lower surface of the deepest leaves [85], which, in the case of dense canopies, is difficult to cover with insecticides [88]. Therefore, the effectiveness of contact insecticides has to be carefully considered, and the application must be adapted to achieve a good coverage on both the well-exposed leaves and on basal leaves located within the canopy [87].

The egg-lying behavior of the vectors must also be considered in IPM control, for example, *S. titanus* laying overwintering eggs under the bark of permanent wood and fruiting shoots from the previous growing season. These are usually removed during winter pruning [89]. Because *S. titanus* can occasionally lay eggs on one-year-old wood too, if these are used as grapevine propagative material, they have to be disinfected and carefully controlled before planting [90].

Altogether, the combinations of chemical control using insecticides can reduce vector populations, parallel biological control, such as natural enemies and entomopathogenic fungi, and offer sustainable alternatives for vector management [91].

## 8. Future Perspectives—Precision Agriculture and Smart Monitoring

Precision agriculture and smart monitoring technologies are transforming plant disease management by enabling real-time, data-driven decision-making. In phytoplasma pathosystems where the early detection of both infected plants and vector populations is critical, these approaches provide powerful tools to improve surveillance, optimize interventions, and reduce unnecessary inputs [92].

### 8.1. Monitoring of Vector Populations

The accurate and timely monitoring of insect vectors is essential for predicting phytoplasma spread and timing control measures. Precision agriculture integrates sensor-based traps, remote sensing technologies, and automated data collection systems to track vector abundance and activity. Smart traps equipped with optical sensors or imaging systems can detect and quantify insect populations in real time, reducing reliance on manual sampling. These systems can be combined with machine learning algorithms for species identification and population trend analysis, improving the precision of vector management [93,94].

### 8.2. Detection of Plant Infection

The early detection of phytoplasma-infected plants is challenging due to latent infection periods and non-specific symptoms. Precision agriculture addresses this limitation through advanced sensing technologies, including hyperspectral and multispectral imaging, thermal imaging, and fluorescence-based sensors [95].

These tools can detect subtle physiological and biochemical changes in plants before visible symptoms appear. Remote sensing platforms, including drones and satellite systems, enable large-scale monitoring of crop health, facilitating early intervention [96].

### 8.3. Data Integration and Decision Support Systems

A key advantage of precision agriculture is the integration of heterogeneous data sources into decision support systems (DSSs). These systems combine vector monitoring data, environmental parameters, and crop health indicators to model disease risk and optimize management strategies [97].

Predictive models can forecast vector population dynamics and phytoplasma spread, allowing for timely and targeted interventions. Recent advances in artificial intelligence and big data analytics further enhance DSS capabilities, enabling adaptive and site-specific management [98,99].

## 9. Conclusions

Phytoplasma diseases continue to pose a serious threat to global agriculture due to their complex transmission by insect vectors. Severe diseases can occur, and expansion (i.e., the expansion of the American grapevine leafhopper (*S. titanus*) in Europe) underlines the necessity of developing new and innovative technologies for Phytoplasma control. Traditional chemical control methods are increasingly insufficient due to resistance development and environmental concerns. Innovative approaches such as genome editing, paratransgenesis, semiochemical-based control, nanotechnology, biological control, and precision agriculture technologies provide promising alternatives for sustainable pest management. Integrating these advanced strategies into comprehensive IPM programs offers the best prospects for controlling phytoplasma insect vectors and minimizing crop losses in the future. Altogether, timely and effective assessment and integrated control will be necessary against phytoplasma vectors. Several recent issues in Europe, such as the expansion of the Flavescence dorée phytoplasma in grapevine spread by the *S. titanus* vector, require urgent interventions and further studies on vector control.

Altogether, several comparative assessments, including conventional and modern innovative strategies for phytoplasma insect vector management, can be identified, and their efficiency can be evaluated through the presented factors, including efficacy, sustainability, regulatory maturity, target specificity, and principal implementation challenges (Table 1).

## Figures and Tables

**Table 1:** Comparative assessment and overview of conventional and modern innovative strategies for phytoplasma insect vector management, including efficacy, sustainability, regulatory maturity, target specificity, and principal implementation challenges.

Control Strategy	Mechanism	Efficacy	Sustainability	Regulations	Targets	Challenges
Chemical control	Direct suppression of vectors through neurotoxins and/or physiological disruption	Moderate to high short-term efficacy, declining due to resistance development	Low to moderate due to environmental contamination and non-target impacts	Widely approved and commercially available, but increasingly restricted in many countries	Low to moderate; often affects non-target arthropods	Resistance evolution, environmental contamination, pollinator decline, repeated applications required
Genetic control, genome editing	Replacement of vector populations through engineered genetic modifications	Potentially high if rapid spread is achieved	High because of species-specific and self-propagating effects	Mostly experimental; strict regulatory and ethical issues	Very high due to species- and gene-specific targeting	Regulatory approval, ecological risks, public acceptance, resistance to gene drives, technical limitations for use
RNAi and pathogen-interference	Silencing genes involved in vector competence and/or pathogen persistence	High potential but mostly under controlled conditions	High if delivery systems minimize off-target effects	Limited commercial approvals for agricultural use	High molecular specificity	Efficient field delivery, RNA stability, production cost, off-target concerns
Sterile Insect Technique (SIT)	Population suppression by release of sterile males	Moderate to high in both isolated and open field systems	High because of non-polluting and species-specific interactions	Established for several pests, but not operational for phytoplasma vectors	Very high	Rearing difficulties reduced male competitiveness, high operational costs. No data about sterile male infection rate
Semiochemical-based behavioral control	Manipulation of the vector behavior using pheromones or plant volatiles	Moderate; highly effective when integrated into IPM	High due to reduced pesticide dependence	Partially commercialized for some insect systems	High to moderate, depending on compound specificity	Environmental variability, formulation stability, limited knowledge of vector signaling mechanisms
Nanotechnology-based crop protection	Targeted delivery of insecticides, RNAi, or antimicrobial compounds using nanoparticles	Moderate to high potential with improved delivery precision	Moderate to high, depending on nanoparticle composition and persistence	Early-stage regulatory frameworks; limited agricultural standardization and use	Moderate to high depending on formulation	Environmental problems, toxicity assessment, production cost, regulatory issues
Symbiont manipulation	Modification of insect-associated microbiota to reduce vector fitness	Promising but still largely experimental	High potential due to biological self-maintenance	Experimental with limited field validation	High because symbionts are vector-associated	Stable symbiont engineering, ecological unpredictability, regulatory barriers
Biological control (parasitoids, predators, entomopathogenic fungi)	Natural suppression of vector populations	Moderate; variable under field conditions	Very high due to ecological compatibility	Widely accepted and increasingly promoted in sustainable agriculture	Moderate to high, depending on biological agent	Environmental sensitivity, inconsistent establishment in field conditions
Conservation, biological control, and habitat management	Enhancement of natural enemy activity through ecological engineering	Moderate but effective long-term	Very high	Highly compatible with sustainable agriculture policies	Moderate	Requires landscape-level planning, delayed results, dependence on local biodiversity
Precision agriculture and smart monitoring	Real-time detection and targeted management using sensors	Indirect but highly effective for early intervention	High due to reduced chemical inputs	Increasingly adopted with few regulatory limitations	High spatial and temporal precision	High initial investment, technical expertise, data integration requirements
Integrated Pest Management (IPM)	Combination of biological, chemical, behavioral approaches	High when multiple compatible strategies are integrated	Very high, as it minimizes reliance on single interventions	Globally promoted and supported by agricultural policy frameworks	Variable depending on combined methods	Requires coordination, monitoring infrastructure, and multidisciplinary management

## Data Availability

No new data were created or analyzed in this study. Data sharing is not applicable to this article.
